# Exosomal miR-7-25207 Increases Subgroup J Avian Leukosis Virus Titers by Targeting the Akt-CyclinQ1 and PRC1-YAF2 Dual Pathways

**DOI:** 10.3390/microorganisms12071495

**Published:** 2024-07-22

**Authors:** Xiaona Zeng, Tongfei Liu, Shengqiu Tang, Xiaoying Dong, Yajuan Li, Liqin Liao, Sheng Chen, Liyi Chen, Jie Kong, Zhenkai Dai, Keyu Feng, Yung-Hou Wong, Qingmei Xie

**Affiliations:** 1 State Key Laboratory of Swine and Poultry Breeding Industry & Heyuan Branch, Guangdong Provincial Laboratory of Lingnan Modern Agricultural Science and Technology, College of Animal Science, South China Agricultural University, Guangzhou 510642, China; xiaona.zeng@scau.edu.cn (X.Z.); 13360001482l@163.com (T.L.); lyj119963@163.com (Y.L.); lapchin_l@outlook.com (L.L.); chens@stu.scau.edu.cn (S.C.); lychen@stu.scau.edu.cn (L.C.); dreamgirl0623@scau.edu.cn (J.K.); zhenkai.dai@uqconnect.edu.au (Z.D.); fky19842004@163.com (K.F.); 2 Henry Fok School of Biology and Agriculture, Shaoguan University, Shaoguan 512005, China; willertang@163.com (S.T.); dongcathy@163.com (X.D.); 3 Guangdong Provincial Key Lab of Agro-Animal Genomics and Molecular Breeding, College of Animal Science, South China Agricultural University, Guangzhou 510642, China; 4 Guangdong Engineering Research Center for Vector Vaccine of Animal Virus, Guangzhou 510642, China; 5 Division of Life Sciences, Biotechnology Research Institute, Hong Kong University of Science and Technology, Hong Kong, China; boyung@ust.hk

**Keywords:** subgroup J avian leukosis virus, exosome, replication, miR-7-25207, Akt-CyclinQ1 pathway, PRC1-YAF2 pathway

## Abstract

Subgroup J avian leukosis virus (**ALV-J**) is a major pathogen in poultry, causing substantial economic losses to the poultry industry worldwide. Exosomal small RNAs derived from virus-infected cells or biological fluids can serve as viral transmission vectors. However, the role and mechanism of exosomal miRNA in ALV-J infection are unclear. In this study, we demonstrated that exosomal microRNA-7-25207 (**miR-7-25207**) could increase the titers of ALV-J. Exosomes isolated from ALV-J-infected DF-1 cells (**Exo-ALV-J**) contained partial viral proteins from ALV-J and could transmit the infection to uninfected DF-1 cells, leading to productive infection. Additionally, the RNA expression profile of exosomes was altered following ALV-J infection. miRNA analysis revealed that the expression of exosomal miR-7-25207 increased. Overexpression of miR-7-25207 significantly increased the titers of ALV-J in transfected cells. Furthermore, miR-7-25207 directly suppressed the expression of Akt and PRC1. Akt, in turn, directly inhibited CyclinQ1 expression, while PRC1 directly interfered with YAF2 expression. In conclusion, ALV-J infection activates the expression of miR-7-25207, which is subsequently delivered via exosomes to uninfected cells, increasing ALV-J titers by targeting Akt-CyclinQ1 and PRC1-YAF2 dual pathways. These findings suggest that exosomal miR-7-25207 may serve as a potential biomarker for clinical parameters in ALV-J infection.

## 1. Introduction

Subgroup J avian leukosis virus (**ALV-J**) is a highly oncogenic single-stranded RNA retrovirus that can lead to growth inhibition, visceral neoplasms, and severe immunosuppression in poultry flocks, causing significant economic losses within the worldwide poultry industry [1,2,3,4]. The ALV-J genome has an organization consisting of gag–pro–pol–env, which encodes the capsid protein p27 (an antigen common to all subgroups of ALV) [5,6,7,8]. The pathogenesis of ALV-J has not been completely elucidated. It has been reported that ALV-J infection can promote the production of exosomes and that exosomes mediate both the horizontal and vertical transmission of ALV-J [8,9].

Exosomes are cup-shaped and double-membraned vesicles with a diameter of 50 to 150 nm, which participate in various biological and pathological processes [10]. Exosomes carry various cargos such as proteins, lipids, nucleic acids (mRNA, miRNAs, and DNA), and metabolites, with the specific cargos varying in different cell lines. Special sequences are required to sort cargo mRNA or miRNAs in exosomes [11,12]. miRNAs in exosomes play an important role in the cancer process. For instance, exosomes contain anti-metastasis cargos, such as miR-23b [13]. Moreover, exosomes from pancreatic cancer possess increased levels of miRNAs to prepare pre-metastatic niches in the lungs or lymph nodes for tumor cell hosting [14]. As a vector for the transmission of various viruses, exosomes also play a significant role in the pathogenesis of viruses, such as ALV-J [2], zika virus [15], classical swine fever virus [16], and porcine reproductive and respiratory syndrome virus [17]. Still, the role of exosomes in the pathogenesis of the infection remains poorly understood. miRNA is a class of small regulatory RNAs that play crucial roles in various biological processes, including cell proliferation, cell death, cell development and differentiation, viral infection, hematopoiesis, and tumor formation [18,19,20]. Although the field of exosomes has rapidly developed, we still do not fully understand the detailed regulation and function of exosomes, especially exosomal miRNAs. Continued studies in exosomal miRNAs will bring new insights into the role of intercellular communications in various biological functions and disease progression.

The role of exosomal miRNA in ALV-J pathogenesis should not be underestimated. To explore this, we investigate the true impact and potential mechanisms of exosomal miRNA on the pathogenesis of the ALV-J infection.

## 2. Materials and Methods

### 2.1. Viruses and Cells

ALV-J viruses (the GD1109 strain, GenBank accession no. JX254901.1) used in this study were associated with hemangioma and stored in our lab in South China Agricultural University. DF-1 cells (immortalized chicken embryo fibroblast cells) were cultured in DMEM (Thermo Fisher Scientific, Inc., Waltham, MA, USA) supplemented with 10% exosome-depleted FBS (System Biosciences, Inc., Palo Alto, CA, USA) and 1% penicillin–streptomycin and incubated at 37 °C with 5% CO_2_.

### 2.2. Isolation and Purification of Exosome

Exosome samples were collected from ALV-J-infected (**Exo-ALV-J**) and uninfected DF-1 cells (**Exo-DF**), respectively. A two-step (ultracentrifuge and immunomagnetic) method was used for the isolation of Exo-ALV-J and Exo-DF according to the procedure previously described [2]. Briefly, supernatants from cultures of ALV-J-infected and uninfected DF-1 cells were collected and centrifuged (300× *g*, 10 min, 4 °C; 200× *g*, 10 min, 4 °C; 10,000× *g*, 30 min, 4 °C) to remove cellular debris. Subsequently, supernatants were ultracentrifuged (100,000× *g*, 70 min, 4 °C; washed in PBS; 100,000× *g*, 70 min, 4 °C) to collect the exosome mixture. The resulting exosome mixture was resuspended in PBS. Then, the Exo-ALV-J, Exo-DF, and free ALV-J in the exosome mixture were further separated using an Exosome–human CD63 Isolation kit (exosome marker protein CD63-labeled-Dynabeads; Invitrogen; Thermo Fisher Scientific, Inc., USA) and purified using an Exo-Quick kit (Along Technology, Beijing, China) according to the manufacturer’s protocol [2]. Briefly, add 50 μL isolation buffer into 50 μL resuspended exosome mixture. Resuspend the magnetic beads by vertexing for 30 s. Wash the magnetic beads by adding 500 μL of isolation buffer. Place the tube on the magnet for 1 min and discard the supernatant. Add isolation buffer (100 μL final volume) to the magnetic beads and incubate the tube overnight at 2 °C to 8 °C. Centrifuge the tube for 3–5 s to collect the sample at the bottom of the tube. Wash the bead-bound exosomes by adding 300 μL of isolation buffer. Place the tube on the magnet for 1 min and discard the supernatant. Add 400 μL of isolation buffer. Place the tube on the magnet for 1 min and discard the supernatant. After isolation and purification, Exo-ALV-J and Exo-DF were separated from free ALV-J virion and used for downstream experiments.

### 2.3. Electron Microscopy and Nanoparticle Tracking Analysis (NTA)

The purified Exo-DF or Exo-ALV-J was resuspended in PBS, spotted onto formvar-coated 400-mesh copper grids, fixed in 4% paraformaldehyde at 25 °C for 30 min, stained with urany1 acetate for 1 min, and visualized under a transmission electron microscope (**TEM**; Talos F200S; Thermos Fisher Scientific, Inc., USA).

NTA was performed using a Nano-Sight LM-10 instrument (Nano-Sight, Spectris, Shanghai, China) following the manufacturer’s procedure. Each sample of Exo-DF or Exo-ALV-J was subjected to three 1 min NTA treatments. The particle size distribution and concentration for each sample were obtained by analyzing a 1 min video for data collection using NTA software (version 2.3).

### 2.4. Exosome Labeling and Uptake

Exo-DF or Exo-ALV-J was labeled with the red dye DiI (1,1′-Dioctadecyl-3,3,3′,3′-tetramethylindocarbocyanine perchlorate; Sigma Aldrich; Merck KGaA, Burlington, MA, USA) following the manufacturer’s procedure. Subsequently, DiI-labeled Exo-DF or Exo-ALV-J (25 mg/mL) were added into DF-1 cells (1 × 10^5^) and incubated at 37 °C for 2 h. After incubation and three times washing with PBS, cells were incubated and labeled with green fluorescent DiO (3, 3′-Dioctadecyloxacarbocyanine Perchlorate; Sigma-Aldrich; Merck KGaA, USA) at 37 °C for 2 h. After incubation and three times washing with PBS, imaging was performed to detect the localization of Exo-DF or Exo-ALV-J in DF-1 cells using a fluorescence-inverted microscope (Nikon, Inc., Tokyo, Japan).

### 2.5. Infection Assay and Immunofluorescence Assay

For infection assays, DF-1 cells were seeded at a density of 1 × 10^5^ cells per well in a 12-well plate. Exo-DF (25 mg/mL), Exo-ALV-J (25 mg/mL), or ALV-J (5 × 10^5^ TCID_50_) were added to the cells. After 2 h of incubation, the cell-cultured medium was replaced with a fresh medium and incubated at 37 °C for 48 h. At 48 h post-infection, cell samples were collected, and total RNAs were extracted to determine ALV-J RNA copy numbers by qRT-PCR. At the same time, the collected cell samples were used to detect ALV-J antigen titers by ELISA and to analyze ALV-J-p27 protein expression by Western blotting.

For immunofluorescence assay, at 48 h post-infection, cells were labeled using a green fluorescent DiO (3,3′-Dioctadecyloxacarbocyanine Perchlorate; Sigma-Aldrich; Merck KGaA, USA) to stain the plasma membranes of the cells. After three times washing with PBS, cells were fixed with 4% paraformaldehyde, permeabilized, blocked with 5% BSA solution, and incubated with a primary antibody. After incubation and three times washing with PBS, cells were immunofluorescence stained with Alexa Fluor 594-conjugated anti-mouse IgG secondary antibody (Thermo Fisher Scientific, Inc., USA). The cell nuclei were stained with DAPI (4′,6-Diamidino-2-phenylindole; Sigma-Aldrich; Merck KGaA, USA). Subsequently, imaging was performed to detect the localization of ALV-J-gp85 in DF-1 cells using a fluorescence-inverted microscope (Nikon, Inc., Japan). For the immunofluorescence assay, the primary antibody used was monoclonal ALV-J-gp85 (made in our laboratory) [2].

### 2.6. Total RNA Extraction, RT-PCR, and qRT-PCR Assay

Total RNAs were extracted from DF-1 cells infected with Exo-DF, Exo-ALV-J, or ALV-J using TRIzol^®^ reagent (Thermo Fisher Scientific, Inc., USA) according to the manufacturer’s protocol. RNA integrity was assessed using the RNA Nano 6000 Assay Kit of the Bioanalyzer 2100 system (Agilent Technologies, Santa Clara, CA, USA). Next, RNA was reverse-transcribed into cDNA using a Prime-Script™ cDNA synthesis kit (Takara Bio, Inc., Beijing, China). RT-PCR was performed on BIO-Rad (Bio-Rad Laboratories, Inc., Hercules, CA, USA) following the manufacturer’s procedure. A 150 bp fragment of gene ALV-J-p27 was amplified using the primer pairs ALV-J-p27 (Table 1). In addition, qRT-PCR was performed on the CFX96 system (Bio-Rad Laboratories, Inc., USA) using Power SYBR Green PCR Master Mix (Roche Diagnostic, Indianapolis, IN, USA) following the manufacturer’s procedure. The mRNA levels of ALV-J were measured by qRT-PCR. Gene glyceraldehyde-3-phosphate dehydrogenase (**GAPDH**) was used as the internal reference with primer pairs GAPDH (Table 1). Gene relative expression was calculated using the comparative 2^−ΔΔCT^ method.

### 2.7. Western Blot Analysis

Exosome samples were lysed in radioimmunoprecipitation assay buffer (**RIPA**; Santa Cruz Biotechnology, Inc., Santa Cruz, CA, USA), loaded and resolved by sodium dodecyl sulfate–polyacrylamide gel electrophoresis (**SDS-PAGE**), and transferred to polyvinylidene fluoride membranes (**PVDF**; EMD Millipore, Burlington, MA, USA). Next, the PVDF membranes were blocked in 5% skimmed milk and 0.05% Tween 20 in PBS, incubated with primary antibodies at 4 °C overnight. Subsequently, the PVDF membranes were incubated with a secondary peroxidase-conjugated antibody (Beyotime Institute of Biotechnology, Haimen, China) for 1 h at room temperature. Chemiluminescence was detected using an enhanced chemiluminescence kit (CW Biotechnology, Taizhou, China), and bands were imaged using an Azure c300 digital imager system (Azure Biosystems, Dublin, CA, USA). Primary antibodies used were glucose-regulated protein 78 kD (**GRP78**; Abcam, Cambridge, UK), β-actin (CW Biotechnology, China), cluster of differentiation 81 (**CD81**; System Biosciences, Inc., Palo Alto, CA, USA), and tumor susceptibility gene 101 (**TSG101**; Absin Biotechnology, Shanghai, China).

### 2.8. miRNAomics Assay

Total RNA was extracted from the Exo-DF, Exo-ALV-J, and DF-1 cells using the RNeasy Plant Mini kit (Qiagen, Hilden, Germany) according to the protocol. RNA concentration was measured at 260 nm. RNA purity was verified using the Nanophotometer^®^ spectrophotometer (IMPLEN, Westlake Village, CA, USA). RNA integrity was assessed using the RNA Nano 6000 Assay Kit of the Bioanalyzer 2100 system (Agilent Technologies, CA, USA). A total of 1 µg RNA per sample was used as input material for miRNA sequencing on the Illumina^®^ HiSeq2000 system (San Diego, CA, USA). The performed steps included rRNA removal, RNA fragmentation, double-stranded cDNA synthesis, adenylated-end addition, adapter addition, PCR amplification, library-quality test, and sequencing on next-generation sequencing NextSeq550 platforms. Clean reads were obtained by removing the raw reads of RNA-seq with adaptors. The quality of the clean reads was analyzed using the software FastQC v.011.9. Furthermore, the annotation information, relative abundance tables, principal component analysis (**PCA**), Kyoto Encyclopedia of Genes and Genomes (**KEGG**) homolog spectra, and pathway maps were performed based on the online miRNA annotation database (miRbase, https://www.mirbase.org, accessed on 19 December 2023). The threshold was selected as *p* < 0.05.

### 2.9. miR-7-25207 Overexpression and Interference

The miR-7-25207 mimics, miR-7-25207 inhibitors, and corresponding negative control were constructed by Shenggong (Shanghai, China). The sequences for miR-7-25207 mimics were 5′-UGCCCAUAAACCAUAACUGUGG-3′ and 5′-ACAGUUAUGGUUUAUGGGCAUU-3′, while the sequence for miR-7-25207 inhibitor was 5′-CCACAGUUAUGGUUUAUGGGCA-3′. miR-7-25207 mimics or inhibitors (2 μg/well) were transfected into a 6-well plate (seeded with 1 × 10^5^ DF-1 cells) using Lipofectamine 3000 (Thermo Scientific, Waltham, MA, USA) following the manufacturer’s instructions. At 24 h post-transfection, cells were infected with ALV-J (5 × 10^5^ TCID_50_). At 48 h post-infection, cell-culture supernatants were collected for ELISA assay, and cells were collected for qRT-PCR and Western blot assay. For qRT-PCR, the predicted genes targeted by miR-7-25207 were measured using the primers, as shown in Table 1. For the Western blot assay, the primary antibody used was monoclonal ALV-J-p27 (made in our laboratory).

### 2.10. ELISA Assay

The antigen titers of ALV-J were detected using an ALV-J-specific ELISA antigen detection kit (NECVB, Harbin, China) following the manufacturer’s instructions. A total of 100 μL per well of the sample was added to the ELISA plate and incubated at 37 °C for 1 h. Subsequently, 300 μL per well of wash solution was added and the plate was washed four times. 100 μL per well of antibody was added to the ELISA plate and incubated at 37 °C for 1 h, followed by another four washes. A total of 50 μL per well of substrate solution was added and incubated at 25 °C for 15 min. Finally, 50 μL per well of termination solution was added, and readings were taken at a wavelength of 630 nm.

### 2.11. Data Availability

Raw reads of miRNA-Seq in this study have been deposited in the NCBI database (https://www.ncbi.nlm.nih.gov/geo/) accessed on 11 October 2023 under the accession number PRJNA1025914.

### 2.12. Statistical Analysis

GraphPad Prism 8.0 (GraphPad Software, Inc., San Diego, CA, USA) was used for statistical analysis. The results represent the mean ± standard error of the mean. Differences among multiple groups were assessed using ANOVA analysis.

## 3. Results

### 3.1. Identification of Exosome Morphology and Protein Expression

**Figure 1:**
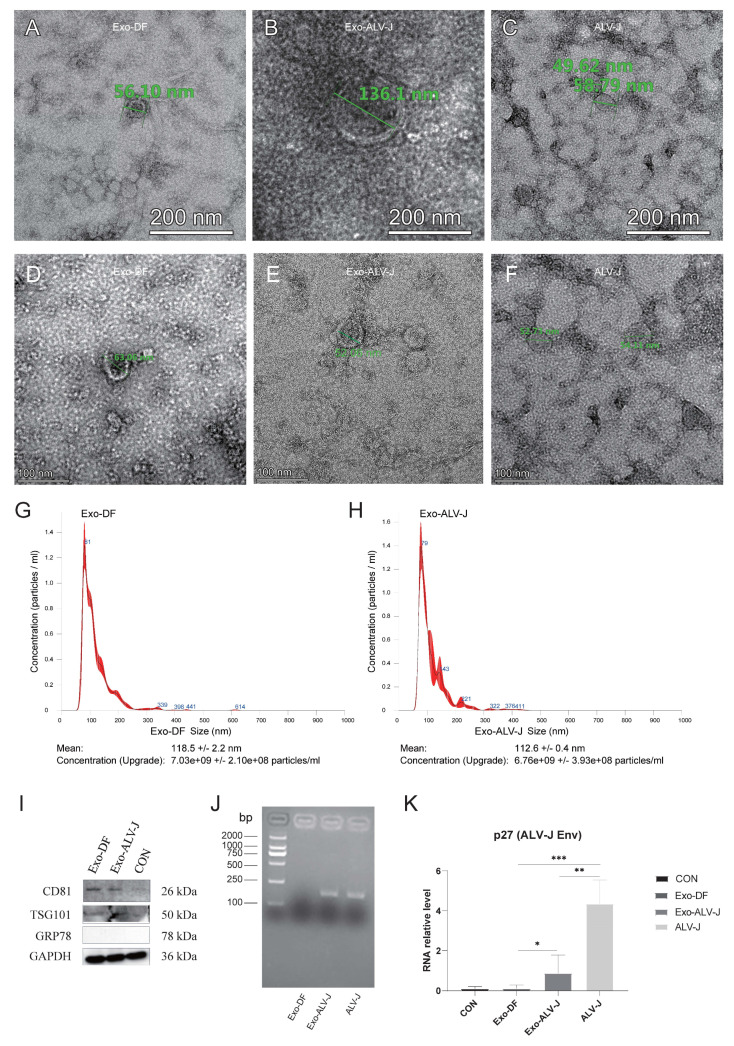
Isolation and characterization of exosomes derived from ALV-J-infected or uninfected cells. (**A**–**F**) Transmission electron microscopy observations of exosomes from ALV-J-uninfected cells (Exo-DF) (**A**,**D**), ALV-J-infected cells (Exo-ALV-J) (**B**,**E**), and free ALV-J (**C**,**F**). The scale bar indicates 200 nm and 100 nm in (**A**–**C**), and (**D**–**F**), respectively. (**G**,**H**) Size distribution and concentration of Exo-DF (**G**) and Exo-ALV-J (**H**) determined by NTA. Mean represents the mean size. Concentration represents the particle concentration. (**I**) Western blot analysis on Exo-ALV-J and Exo-DF. Results use antibodies against the common exosome markers (CD81 and TSG101), and the endoplasmic reticulum marker GRP78. (**J**) Exo-ALV-J was confirmed to contain ALV-J genomic RNA using RT-PCR detection for the ALV-J p27 gene. (**K**) Real-time qPCR analysis (qRT-PCR) demonstrated productive infection of DF-1 cells after being infected with Exo-ALV-J. The *y*-axis shows the relative level of ALV-J p27 gene expression. *, *p* < 0.05. **, *p* < 0.01. ***, *p* < 0.001. Exo-DF, exosome derived from ALV-J-uninfected cells. Exo-ALV-J, exosome derived from ALV-J-infected cells. CON, purified ALV-J particles were used as control.

**Figure 2:**
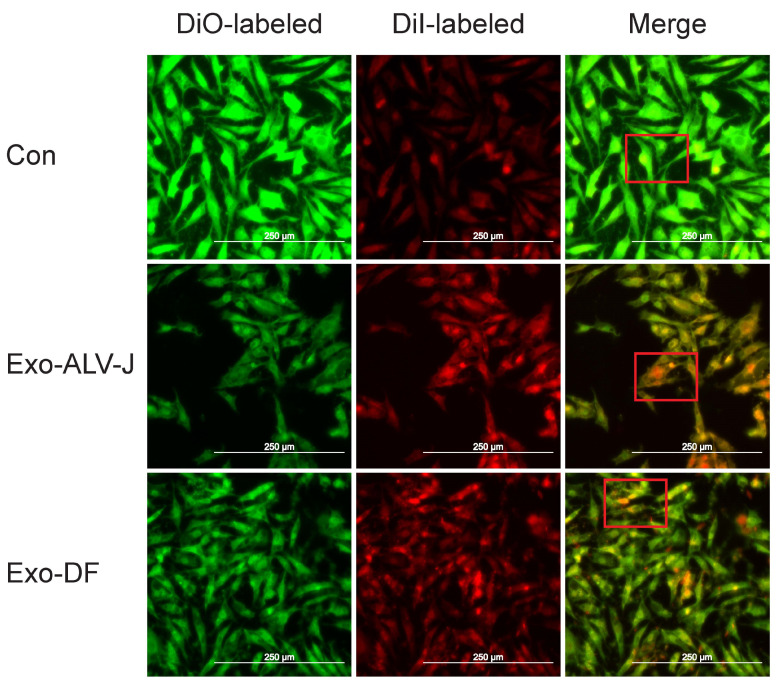
Exosome uptake assay showed that both Exo-ALV-J and Exo-DF can be uptaken by DF-1 cells. Exosome Exo-ALV-J and Exo-DF were first stained with the red fluorescent dye DiI for 20 min and then added to DF-1 cells for incubation. After 2 h of incubation at 37 °C, DF-1 cells were fixed and stained with the green fluorescent dye DiO. The merged image shows the co-localization of DiO-labeled DF-1 cells incubated with DiI-labeled exosome Exo-ALV-J or Exo-DF. The merged image in the red box shows the discernible distinction between CON, Exo-ALV-J, and Exo-DF. The scale bar indicates 250 μm. Exo-DF, exosome derived from ALV-J-uninfected cells. Exo-ALV-J, exosome derived from ALV-J-infected cells. Con, uninfected DF-1 cells as the negative control.

Exosomes isolated from ALV-J-infected DF-1 cells (**Exo-ALV-J**) or uninfected DF-1 cells (**Exo-DF**) were identified by morphology and the expression of marker proteins. Transmission electron microscopy (**TEM**) examination and nanoparticle tracking analysis (**NTA**) were used for the morphology verification of exosomes. TEM analysis showed that both Exo-DF (Figure 1A,D) and Exo-ALV-J (Figure 1B,E) exhibit an exosomal classical cup-shaped appearance with a lipid bilayer membrane. Free ALV-J particles display a spherical appearance with an enveloped structure (Figure 1C,F). Moreover, NTA analysis revealed a similar size distribution pattern for Exo-DF (Figure 1G) and Exo-ALV-J (Figure 1H), with over 80% of exosomes observed having sizes ranging from 40 to 200 nm. The average size of Exo-DF was 118 nm, while the average size of Exo-ALV-J was 112 nm (Figure 1G,H). Subsequently, the purified Exo-DF and Exo-ALV-J were characterized by Western blotting analysis. Both Exo-DF and Exo-ALV-J expressed exosome marker proteins CD81 and TSG101, and both lacked expression of GRP78 (an endoplasmic reticulum marker) (Figure 1I), suggesting no contamination with free ALV-J. In addition, both DiI-labeled Exo-ALV-J and Exo-DF were observed to colocalize with the plasma membrane of DiO-labeled DF-1 cells (Figure 2), indicating that exosomes (Exo-ALV-J and Exo-DF) were successfully uptaken by DF-1 cells.

### 3.2. Exo-ALV-J Encapsulates ALV-J and Causes Productive Infection of ALV-J

To validate whether there was an ALV-J genome encapsulated in the exosome Exo-ALV-J, RT-PCR was performed for amplification of ALV-J-p27. The result showed that the gene ALV-J-p27 was successfully amplified from exosome Exo-ALV-J (Figure 1J), indicating that Exo-ALV-J encapsulated the ALV-J genome. Furthermore, the mRNA expression of the ALV-J virus protein p27 in DF-1 cells infected with Exo-ALV-J was significantly higher than that in DF-1 cells infected with Exo-DF (Figure 1K), indicating that Exo-ALV-J could transfer ALV-J components to uninfected DF-1 cells.

**Figure 3:**
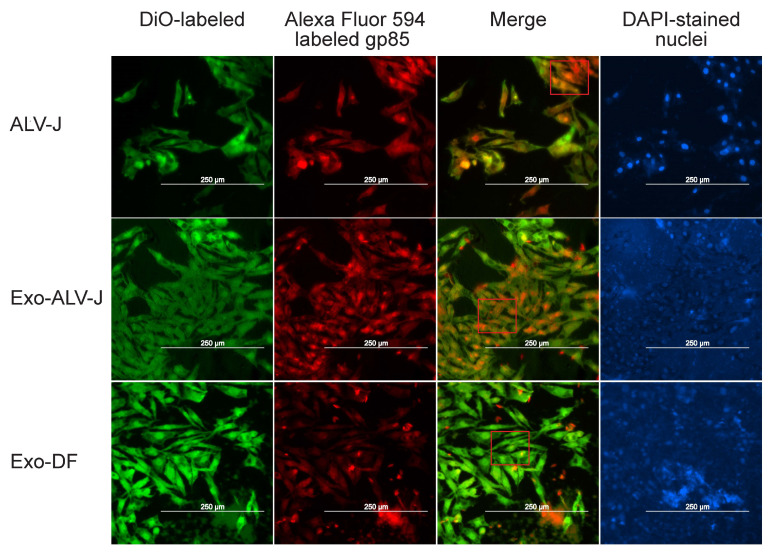
Exo-ALV-J can transmit ALV-J components to uninfected-DF-1 cells. Exo-ALV-J contained ALV-J envelope protein gp85. After being incubated with Exo-ALV-J or Exo-DF for 2 h, DF-1 cells were incubated with an anti-gp85 antibody and then stained with Alexa Fluor 594-conjugated anti-mouse IgG (red). The cell nuclei were stained with DAPI (blue). The merged image shows the co-localization of DiO-labeled DF-1 cells incubated with Exo-ALV-J or Exo-DF and anti-gp85 antibody stained with Alexa Fluor 594-conjugated anti-mouse IgG (red). The merged image in the red box shows the discernible distinction between CON, Exo-ALV-J, and Exo-DF. The scale bar indicates 250 μm. Exo-DF, exosome derived from ALV-J-uninfected cells. Exo-ALV-J, exosome derived from ALV-J-infected cells. Purified ALV-J particles were used as control.

To investigate whether Exo-ALV-J can cause productive infection, uninfected DF-1 cells incubated with Exo-ALV-J, Exo-DF, or ALV-J were used for immunofluorescence assay. The result shows that both Exo-ALV-J and ALV-J could be colocalized in the cytoplasm of DF-1 cells (Figure 3), suggesting that Exo-ALV-J could express ALV-J envelope protein gp85 and cause infection of ALV-J.

These results suggest that Exo-ALV-J could be taken up by DF-1 cells and subsequently transfer ALV-J components to uninfected naïve DF-1 cells, causing productive infection of ALV-J.

### 3.3. ALV-J Activates the Expression of miR-7-25207

**Figure 4:**
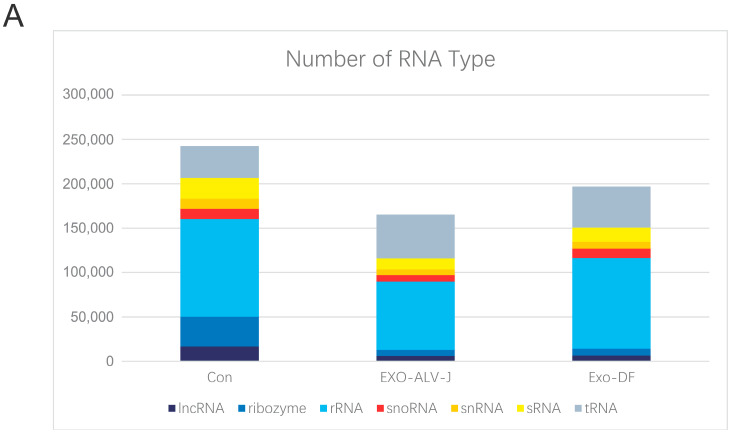

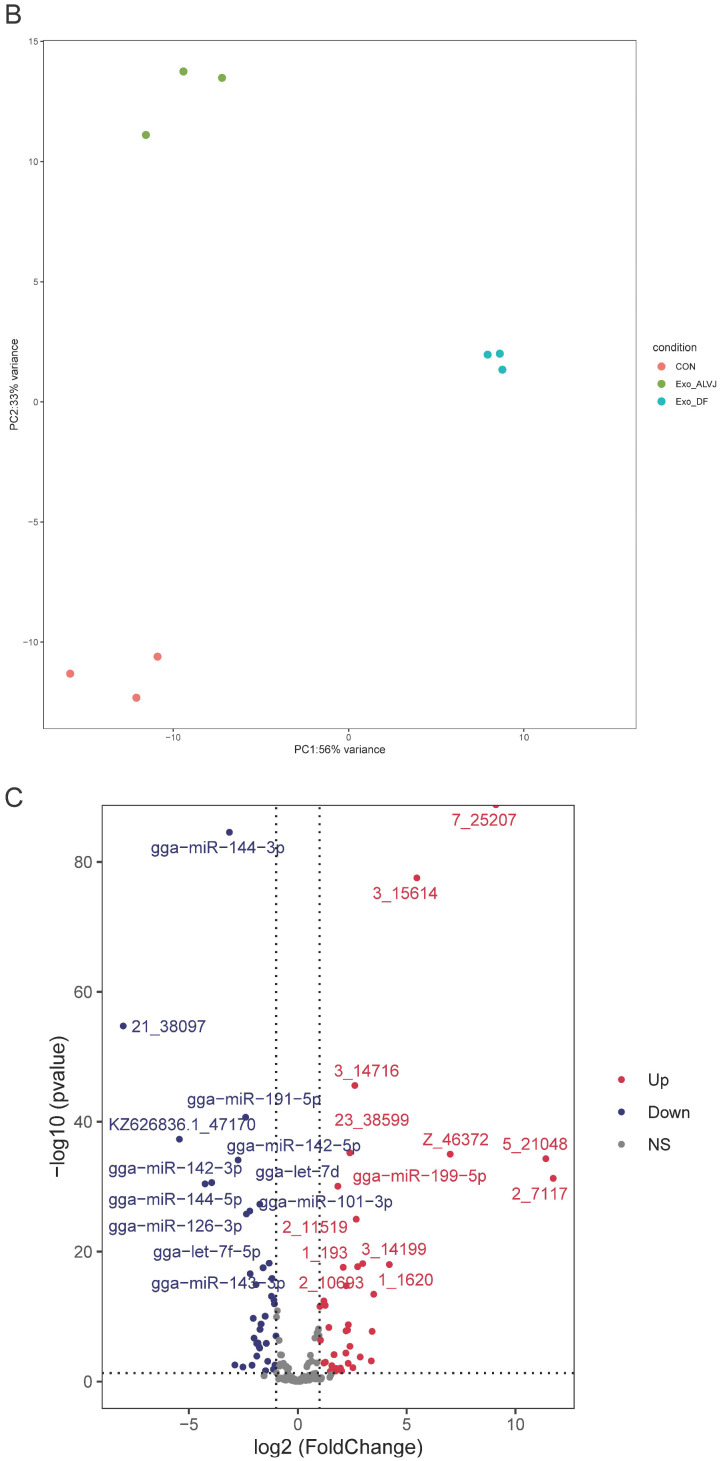
miRNAomics assay of Exo-ALV-J and Exo-DF. (**A**) Numbers of RNA Types of Exo-ALV-J and Exo-DF. Seven types of RNAs were identified in Exo-ALV-J and Exo-DF. The *y*-axis shows the number of RNA sequenced reads. (**B**) Principal component analysis (PCA) of Exo-ALV-J and Exo-DF. PCA identified two clusters in the data separated along the first and second principal components between Exo-ALV-J and Exo-DF. The percentages on each axis represent the percentages of variation explained by the principal components. PC1 and PC2 define 56% and 33% of the variance, respectively. The distance between the points reflects the variance in components between them. (**C**) Volcano plot showing the top 24 differentially expressed miRNAs between Exo-ALV-J and Exo-DF. The volcano plots the distribution of log fold change (*x*-axis) and the negative log (base 10) of the *p*-values (*y*-axis). The data points above the significance threshold (q < 0.05, foldchange > 2) are marked in red (upregulated genes) and blue (downregulated genes).

**Figure 5:**
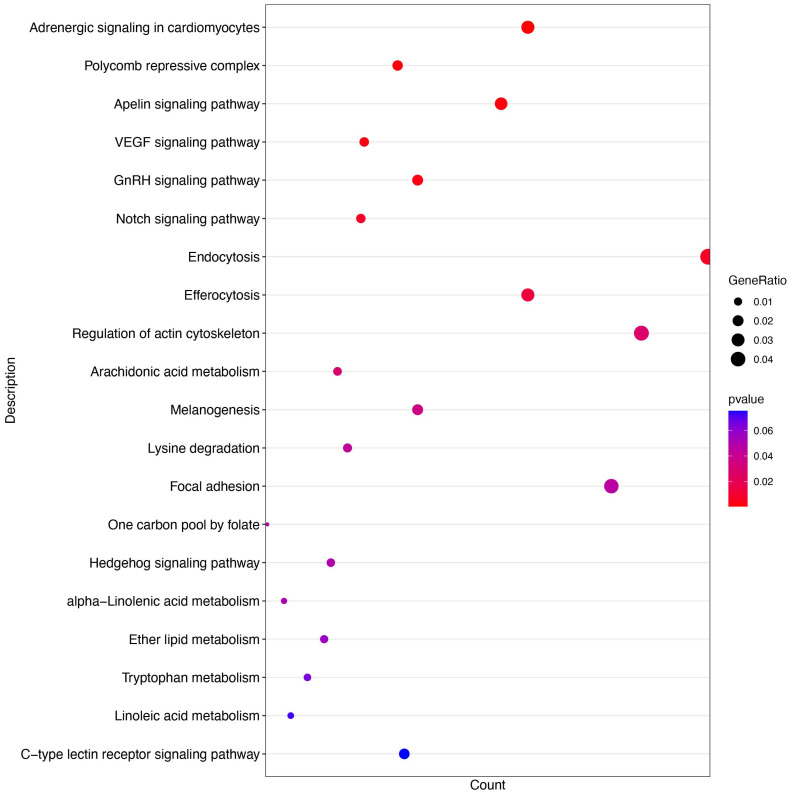
GO analysis of Exo-ALV-J and Exo-DF. Each node represents a GO biological process, the size represents the gene ratio, and the colors represent the *p*-value.

**Figure 6:**
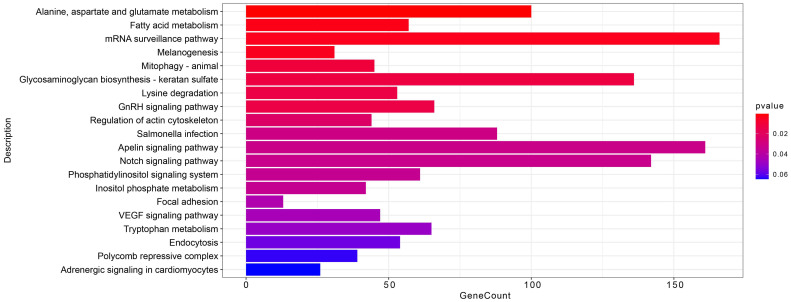
KEGG pathways enrichments of Exo-ALV-J and Exo-DF. Each bar represents a KEGG pathway process, and the colors represent the *p*-value.

A miRNAomics assay was performed to characterize the components of exosomes purified from ALV-J-infected or uninfected DF-1 cells. Seven types of RNAs were identified from Exo-DF and Exo-ALV-J (Figure 4A). rRNA was the most dominant RNA, while snoRNA was the least abundant RNA in the components of Exo-DF and Exo-ALV-J. The abundance of rRNA and long non-coding RNAs (**IncRNA**) was lower in Exo-ALV-J compared with Exo-DF (Figure 4A). A comparison of the principal component analysis (**PCA**) score plot showed different occupied sites in Exo-DF and Exo-ALV-J (Figure 4B). Volcano analysis identified 39 upregulated and 38 downregulated miRNAs in Exo-ALV-J compared with Exo-DF (Figure 4C). miR-7-25207 (Supplementary File S1) was found to be the most upregulated in Exo-ALV-J compared with Exo-DF, while gga-miR-144-3p was the most downregulated (Figure 4C). Gene ontology (**GO**) enrichment analysis revealed differences in cell metabolic processes between Exo-DF and Exo-ALV-J. Categories related to cell projection assembly and protein polymerization were more enriched in Exo-ALV-J compared with Exo-DF (Figure 5). Moreover, Kyoto Encyclopedia of Genes and Genomes (**KEGG**) analysis showed that the metabolites related to adrenergic signaling in cardiomyocytes, polycomb repressive complex, and apelin signaling pathway were more enriched in Exo-ALV-J compared with Exo-DF (Figure 6). The top two KEGG pathways related to cell metabolic processes enriched in Exo-ALV-J were used for further analysis, including polycomb repressive complex signaling pathway (Supplemental File S2A) and apelin signaling pathway (Supplemental File S2B).

### 3.4. miR-7-25207 Increases the Titers of ALV-J

**Figure 7:**
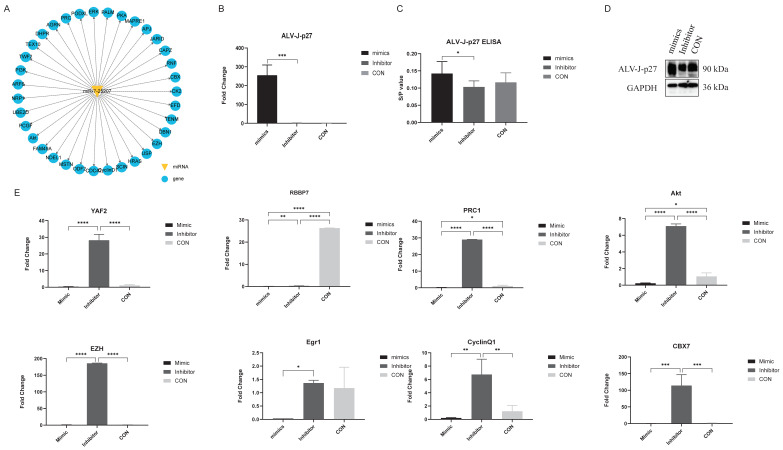
Predicted target genes and qRT-PCR verify assay. (**A**) Predicted downstream target genes of miR-7-25207. Genes marked in blue are potential target genes of miR-7-25207. (**B**). qRT-PCR was performed to detect the expression of ALV-J-p27 in DF-1 cells after transfection. (**C**) ELISA assay was performed to detect viral antigen titer in DF-1 cells after transfection. (**D**) Western blot analysis was performed to detect the protein level of ALV-J-p27 after overexpression of miR-7-25207 mimics and inhibitors. Results use antibodies against the env protein of ALV-J-p27. (**E**) qRT-PCR was performed to detect the RNA relative level of predicted downstream target genes of miR-7-25207. Mimics group represents ALV-J-infected DF-1 cells transfected with miR-7-25207 mimics. Inhibitor group represents ALV-J-infected DF-1 cells transfected with miR-7-25207 inhibitor. CON represents ALV-J-infected DF-1 cells. *, *p* < 0.05. **, *p* < 0.01. ***, *p* < 0.001, ****, *p* < 0.0001.

To examine the involvement of miR-7-25207 in ALV-J infection, we overexpressed and interfered with miR-7-25207 in DF-1 cells using mimics and inhibitors. ALV-J mRNA expression was measured by qRT-PCR, and the results show that miR-7-25207 overexpression significantly increased the mRNA levels of ALV-J-p27, suggesting that miR-7-25207 enhances ALV-J infection (Figure 7B). Furthermore, the ALV-J antigen titer was measured by ELISA, and the results show that miR-7-25207 overexpression markedly increased the S/P value, indicating that miR-7-25207 could enhance the ALV-J antigen titer in DF-1 cells (Figure 7C). Additionally, the env protein of ALV-J was measured by Western blot, and the results show that miR-7-25207 overexpression significantly increased the protein level of ALV-J-p27 (Figure 7D). These results indicate that miR-7-25207 could enhance ALV-J infection.

### 3.5. miR-7-25207 Targets the Akt-CyclinQ1 and PRC1-YAF2/CBX7 Dual Pathways

To identify the downstream target genes of miR-7-25207, we utilized online tools (Targetscan, Mirdb, and Pictar) for analysis. The intersection of these tools revealed the most likely target genes for further verification (Figure 7A). In the polycomb repressive complex pathway, Protein Regulator of Cytokinesis 1/2 (**PRC1/2**) emerged as a potential key target gene of miR-7-25207 (Figure 7A). Additionally, in the apelin signaling pathway, Akt kinase (**Akt**) and CyclinD1 were identified as potential key target genes of miR-7-25207 (Figure 7A).

**Figure 8:**
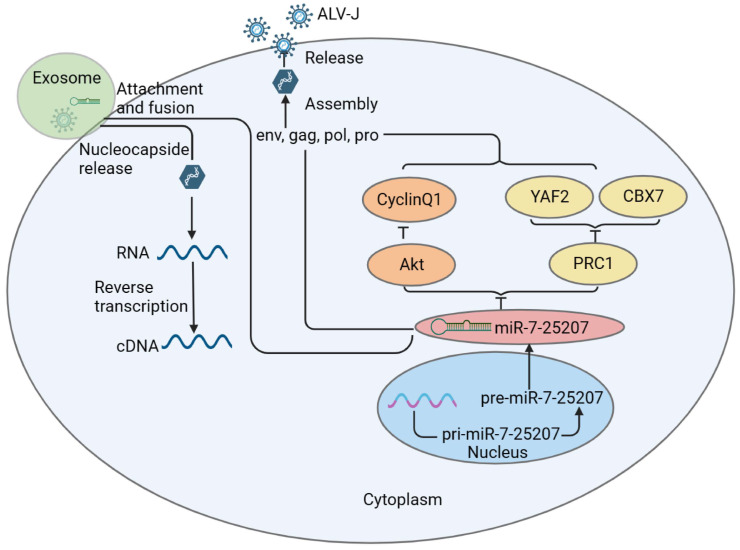
Schematic diagram of the infection and transmission mechanism of ALV-J. Exosomal miR-7-25207 facilitates ALV-J replication by targeting dual pathways of Akt-CyclinQ1 and Prc1-YAF2.

To confirm miR-7-25207 targets, DF-1 cells were transfected with miR-7-25207 mimics or inhibitors, and the expression of the most likely target genes was detected by qRT-PCR. The results show that miR-7-25207 overexpression significantly decreased the mRNA expression levels of YAF2, CyclinQ1, Akt, and PRC1, whereas interfering with miR-7-25207 could upregulate the expression of YAF2, CyclinQ1, Akt, and PRC1 (Figure 7E). These findings suggest that miR-7-25207 targets a dual signaling pathway, Akt-CyclinQ1 and PRC1-YAF2 (Figure 8).

## 4. Discussion

Exosomes are crucial mediators of cell-to-cell communication, facilitating the transfer of biologically active proteins, lipids, and RNAs between cells [21]. Exosomal small RNAs play a significant role in viral pathogenesis, infection, and the survival cycle [22]. Exosomes isolated from ALV-J-infected cells contain partial ALV-J viral proteins and cause productive infections [2]. However, the effect and potential mechanism of exosomal miRNA in ALV-J infection have not been fully elucidated. In this study, we identified a microRNA miR-7-25207 by miRNAomics from exosomes secreted by ALV-J-infected DF-1 cells and explored the relationship between miR-7-25207 and ALV-J infection. Moreover, we found that exosomal miR-7-25207 from ALV-J-infected DF-1 cells enhances ALV-J titers by targeting a dual signaling pathway, Akt-CyclinQ1 and PRC1-YAF2. These findings suggest that exosomal miR-7-25207 may serve as a potential biomarker for clinical parameters in ALV-J infection.

As retroviruses and exosomes share the same secretion pathway [23,24], we used a two-step method (ultracentrifuge and immunomagnetic) to extract and purify exosomes (Exo-ALV-J and Exo-DF) from ALV-J-infected and uninfected DF-1 cells. TEM analysis confirmed that both Exo-DF and Exo-ALV-J displayed the exosome classical cup-shaped appearance with a lipid bilayer membrane. NTA analysis revealed a typical exosome size range of 40–200 nm for both Exo-DF and Exo-ALV-J. The size distribution between Exo-DF and Exo-ALV-J differs. Exo-DF may be mainly composed of microvesicles (~100–1000 nm), while Exo-ALV-J may be primarily exosomes (<150 nm) [2,25,26,27]. Both the purified Exo-DF and Exo-ALV-J were characterized by the expression of exosome marker proteins (CD81 and TSG101) and the absence of GRP78 expression, indicating that both Exo-DF and Exo-ALV-J originated from non-endoplasmic reticulum sources without mixed free ALV-J. These results suggest the effectiveness of the isolation methods in obtaining highly pure Exo-DF and Exo-ALV-J.

Recent studies have shown that the components of exosomes can be altered after viral infection [28]. In this study, the gene ALV-J-p27 was identified in Exo-ALV-J using RT-PCR and qRT-PCR, suggesting the encapsulation of ALV-J viral genomes in Exo-ALV-J. Furthermore, the exosome uptake assay showed that both Exo-ALV-J and Exo-DF were taken up by DF-1 cells. It has been shown that the uptake of exosomes is not random but depends on interactions between proteins on the surface of the exosomes and recipient cells [29,30]. Several reports have suggested that exosomes derived from virus-infected cells can carry and deliver viral genomes, mRNA, proteins, and virions to recipient cells, thereby contributing to viral infection and transmission [31,32]. Moreover, we found that Exo-ALV-J taken up by DF-1 cells could transfer ALV-J genomes to uninfected-DF-1 cells. These results suggested that the components of exosomes were altered after ALV-J infection and that the viral components were packaged into Exo-ALV-J and transmitted to neighboring cells, regulating host cellular behavior and producing productive infections.

Exosomes have been widely studied for their roles in intracellular communication, especially during tumor development. Exosome-associated RNAs, miRNAs, proteins, DNAs, and even metabolites can change the fate of recipient cells by autocrine and paracrine signaling [33]. Due to the heterogeneity of cancer cells, exosomes derived from host cancer cells can activate receptors or alter their miRNA or RNA expression in neighboring cancer cells, thereby altering their biological phenotypes [34,35,36]. Viruses replicate by hijacking host factors, and related factors within infected cells also change to meet the requirements associated with viral replication [37,38]. It has been shown that miRNAs indirectly infect viral replication by regulating key components or directly targeting viral sequences [39]. In this study, a miRNAomics assay was performed to characterize the components and function of exosomes purified from ALV-J-infected or uninfected DF-1 cells. Seven types of RNAs were identified from Exo-DF and Exo-ALV-J. The amount of rRNA and lncRNA were lower in Exo-ALV-J compared to Exo-DF. Growing evidence demonstrated that lncRNA participates in various biological processes by interacting with transcription factors, miRNAs, mRNAs, and RNA-binding proteins to form complicated regulatory networks [40]. IncRNA is implicated in tumor occurrence and progression by regulating the proliferation, differentiation, invasion, and metastasis of cancer cells [41].

Furthermore, PCA score plots showed different occupied sites between Exo-DF and Exo-ALV-J, indicating different expression patterns of RNAs. Subsequently, we identified 39 upregulated and 38 downregulated miRNAs in Exo-ALV-J compared with Exo-DF using volcano analysis, indicating that miRNAs differ in exosomes derived from ALV-J-infected or uninfected cells. Compared with Exo-DF, miR-7-25207 was mostly upregulated, while gga-miR-144-3p was mostly downregulated in Exo-ALV-J. It has been reported that exosome-mediated miR-144-3p could promote ferroptosis to inhibit osteosarcoma proliferation, migration, and invasion [42]. Moreover, miR-144-3p also functions as a tumor suppressor in endometrial cancer. Additionally, miR-144-3p could promote lymph vascular invasion through repression of PTEN/p19 in rectal neuroendocrine tumors [43], regulating the development of various tumors or cancers, such as thyroid tumor [44], lung cancer [45,46], and gastric cancer [47]. miR-144-3p also targeted STC1 to activate the PI3K/AKT pathway, inducing cell apoptosis and cell cycle arrest in selenium deficiency broilers [48].

However, miR-7-25207, as a newly discovered microRNA, has not been reported in related studies. In this study, we confirmed that ALV-J infection can activate the expression of miR-7-25207 and that miR-7-25207 overexpression can facilitate the infection of ALV-J. Furthermore, miR-7-25207 overexpression significantly decreased the mRNA expression levels of YAF2, CyclinQ1, Akt, and PRC1, while interfering with miR-7-25207 could significantly upregulate the expression of YAF2, CyclinQ1, Akt, and PRC1. PRC1 and Akt were identified as the downstream target genes of miR-7-25207. It has been reported that PRC1 complexes are divided into two main groups: canonical PRC1 (**cPRC1**) complexes and non-canonical (**ncPRC1**) complexes. cPRC1 complexes are recruited to chromatin via the H3K27me3 reader activity of CBX proteins. ncPRC1 complexes lack CBX and PHC proteins and instead contain YAF2/RYBP proteins, and their cores can be formed with any of the PCGF1-6 proteins [49,50]. Therefore, we extrapolated that miR-7-25207 increases the titers of ALV-J by targeting the PRC1-YAF2 pathway. In addition, in the apelin signaling pathway, Akt-ERK1/2-CyclinD1 was shown as a signaling pathway related to cell proliferation (Supplemental File S2). Akt is known to be involved in the regulation of cell survival and proliferation by regulating various target molecules [51,52,53]. CyclinQ1, as the downstream target molecule of the Akt signaling pathway, was significantly suppressed after miR-7-25207 overexpression, suggesting that CyclinQ1 was downstream of Akt and suppressed by miR-7-25207. Therefore, we extrapolated that miR-7-25207 increases the titers of ALV-J by targeting the Akt-CyclinQ1 pathway. Taking these results together, we suggest that exosomal miR-7-25207 increases the titers of ALV-J by targeting the Akt/CyclinQ1 and PRC/YAF2 dual pathways.

## 5. Conclusions

In conclusion, this study demonstrated that miR-7-25207 is upregulated by ALV-J and subsequently delivered via exosomes to uninfected cells, increasing ALV-J titers by targeting the Akt/CyclinQ1 and PRC/YAF2 dual pathways. These findings suggest that exosomal miR-7-25207 may serve as a potential biomarker for clinical parameters in ALV-J infections.

## Figures and Tables

**Table 1:** Primers used for gene cloning and expression analysis.

Primer Name ^a^	Primer Sequence (5′-3′)
ALV-J-p27	F: ACAAGACTGGCTGATACGGT
	R: AAGCGTCCATCCATAAGGCA
GAPDH	F: CCCCCATGTTTGTGATGGGT
	R: TGATGGCATGGACAGTGGTC
RBBP7	F: CCTTCTTGTACGACCTGGTGATGAC
	R: GCCAGTGTAGAGCGTAATCCTTTCC
YAF2	F: GGACGAGGGCTACTGGGACTG
	R: ACGAATTGCTGAGGAACCTGCTG
Egr1	F: GACCACTTGACCACGCACATCC
	R: GAGGAAGAAGTTGCTGAGACCGAAG
CyclinD1	F: TCCAGTCTACGCCAGGCACAG
	R: TGTTCACATCTCGCACATCAGTGG
EZH	F: GGGAGGGCTGAACAACGAAACC
	R: CGGCTGTGCTGCTGCTTAGG
CBX7	F: AGGTTTGGAGGAGGAGACAGTAGC
	R: CTTCTTCCTGGACAGTCGCAAGTAC
Akt	F: GTAGCGATAGTGAAGGAAGGATGGC
	R: CGTCTTGCGGTCGTTCCTTGTAG
PRC1	F: CCGCCAGATTGAGACAGAGATGATG
	R: TGGAGAGGGAAGTGCCGTTGAG

^a^ F: forward primer, R: reverse primer.

## Data Availability

The original contributions presented in the study are included in the article/Supplementary Material, further inquiries can be directed to the corresponding author.

## Supplementary Materials

The following supporting information can be downloaded at: https://www.mdpi.com/article/10.3390/microorganisms12071495/s1. File S1: miR-7-25207; File S2: polycomb repressive complex signaling pathway and apelin sig-naling pathway.
