# Supplementary material for: Exosomal miR-7-25207 Increases Subgroup J Avian Leukosis Virus Titers by Targeting the Akt-CyclinQ1 and PRC1-YAF2 Dual Pathways
Source: Microorganisms. 2024 Jul 22;12(7):1495. doi: 10.3390/microorganisms12071495 (PMC11279298; doi:10.3390/microorganisms12071495)
Supplement: Supplementary file 1 [file microorganisms-12-01495-s001.zip › supplemental document S1.pdf]

Provisional ID	:	7_25207
Score total	:	1.9
Score for star read(s)	:	-1.3
Score for read counts	:	0
Score for mfe	:	1.6
Score for randfold	:	1.6
Score for cons. seed	:	
Total read count	:	90074
Mature read count	:	90074
Loop read count	:	0
Star read count	:	0

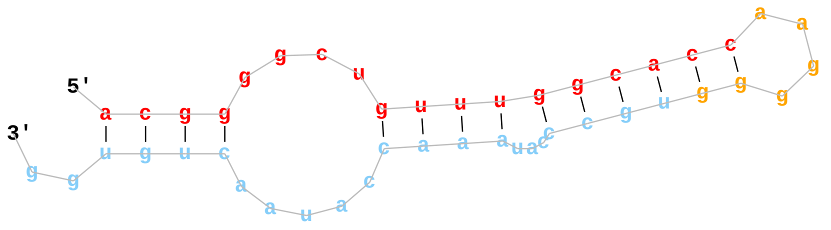

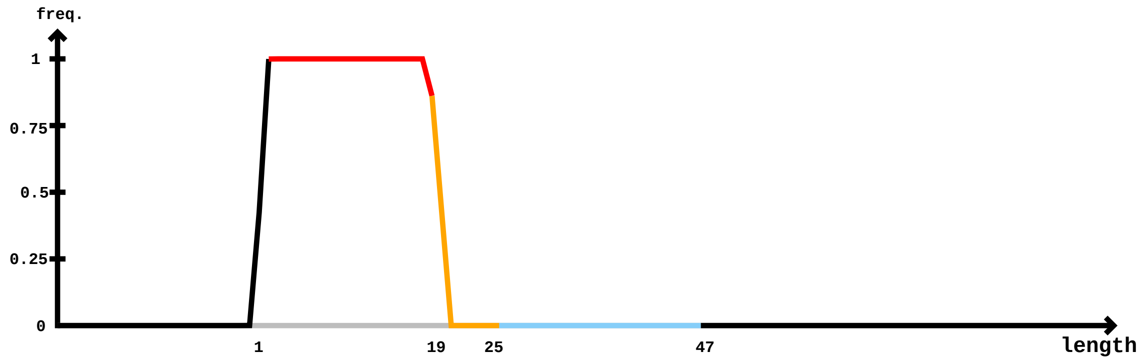

Mature Star

```
5'- gcugugugcuucccagaaugacggggcuguuuggcaccaaggggugcccauaaaccauaacuguggggaagggaugugcagacagaagagcuugcuauccagcucggug -3'   exp
    .((((.(((.((((....(.((((....((((((((((....))))))...)))).....)))).)...))))...))).))))..(((((........))))).....   reads   mm   sample
    ...................gacggggUuguuuggcac.......................................................................   2       1    ED6
    ...................gacggggUuguuuggcacc......................................................................   12      1    ED6
    ....................acggggUuguuuggcacc......................................................................   31      1    ED6

    ...................gacggggUuguuuggcac.......................................................................   5       1    CO3
    ...................gacggggUuguuuggcacc......................................................................   11      1    CO3
    ....................acggggcGguuuggcacc......................................................................   1       1    CO3
    ....................acggggUuguuuggcacc......................................................................   82      1    CO3

    ..................ugacggggUuguuuggca........................................................................   1       1    EA1
    ..................Cgacggggcuguuuggca........................................................................   1       1    EA1
    ..................Cgacggggcuguuuggcacc......................................................................   1       1    EA1
    ..................ugacggggUuguuuggcacc......................................................................   5       1    EA1
    ...................gacggggGuguuuggcac.......................................................................   1       1    EA1
    ...................gacggggUuguuuggcac.......................................................................   3153    1    EA1
    ...................gacggggNuguuuggcac.......................................................................   4       1    EA1
    ...................gacggggcuguuuggcacc......................................................................   1       0    EA1
    ...................gacggggUuguuuggcacc......................................................................   5714    1    EA1
    ...................gacgAggcuguuuggcacc......................................................................   1       1    EA1
    ...................gacggggNuguuuggcacc......................................................................   3       1    EA1
    ...................gacggggUuguuuggcacca.....................................................................   1       1    EA1
    ....................acggggGuguuuggcacc......................................................................   1       1    EA1
    ....................acggggcuguuuggcacc......................................................................   4       0    EA1
    ....................acggggUuguuuggcacc......................................................................   12364   1    EA1
    ....................acgCggcuguuuggcacc......................................................................   1       1    EA1
    .....................cggggUuguuuggcacca.....................................................................   10      1    EA1
    .....................cggggcuguuuggcaccU.....................................................................   4       1    EA1

    ...................gacggggUuguuuggcac.......................................................................   22      1    CO1
    ...................gacggggUuguuuggcacc......................................................................   46      1    CO1
    ....................acggggUuguuuggcacc......................................................................   54      1    CO1
    ....................acggggcGguuuggcacc......................................................................   1       1    CO1

    ...................gacggggUuguuuggcac.......................................................................   96      1    ED1
```

```
                   Mature          Star
gcugugugcuucccagaaugacggggcuguuuggcaccaaggggugcccauaaaccauaacuguggggaagggaugugcagacagaagagcuugcuauccagcucggug
...................gacggggUuguuuggcacc......................................................................  216    1   ED1
....................acggggUuguuuggcacc......................................................................  387    1   ED1

...................gacggggGuguuuggcac.......................................................................  1      1   ED3
...................gacggggUuguuuggcac.......................................................................  100    1   ED3
...................gacggggUuguuuggcacc......................................................................  165    1   ED3
....................acggggUuguuuggcacc......................................................................  373    1   ED3
.....................cggggUuguuuggcacca.....................................................................  2      1   ED3

..................ugacggggUuguuuggca........................................................................  6      1   EA2
..................ugacggggUuguuuggcacc......................................................................  1      1   EA2
...................gacggggNuguuuggcac.......................................................................  4      1   EA2
...................gacggggGuguuuggcac.......................................................................  1      1   EA2
...................gacggggUuguuuggcac.......................................................................  4637   1   EA2
...................gacggggGuguuuggcacc......................................................................  1      1   EA2
...................gacggggcuguuuggcacc......................................................................  7      0   EA2
...................gacggggNuguuuggcacc......................................................................  11     1   EA2
...................gacggggUuguuuggcacc......................................................................  10600  1   EA2
...................gacggggUuguuuggcacca.....................................................................  8      1   EA2
...................gacggggcuguuuggcaccU.....................................................................  2      1   EA2
....................acggggGuguuuggcacc......................................................................  3      1   EA2
....................acggggUuguuuggcacc......................................................................  19372  1   EA2
....................acggAgcuguuuggcacc......................................................................  1      1   EA2
....................acggggcuguuuggcacc......................................................................  2      0   EA2
....................acggggcuguuuggcaccU.....................................................................  2      1   EA2
....................acggggUuguuuggcacca.....................................................................  5      1   EA2
.....................cggggUuguuuggcacca.....................................................................  21     1   EA2
.....................cggggcuguuuggcaccU.....................................................................  3      1   EA2

...................gacggggUuguuuggcac.......................................................................  81     1   ED4
...................gacggggUuguuuggcacc......................................................................  159    1   ED4
....................acggggUuguuuggcacc......................................................................  403    1   ED4
.....................cggggUuguuuggcacca.....................................................................  1      1   ED4

...................gacggggUuguuuggcac.......................................................................  126    1   ED2
...................gacggggUuguuuggcacc......................................................................  259    1   ED2
...................gacggggNuguuuggcacc......................................................................  1      1   ED2
....................acggggUuguuuggcacc......................................................................  455    1   ED2

...................gacggggUuguuuggcac.......................................................................  15     1   CO2
...................gacggggUuguuuggcacc......................................................................  11     1   CO2
....................acggggcGguuuggcacc......................................................................  1      1   CO2
....................acggggUuguuuggcacc......................................................................  30     1   CO2

...................gacggggUuguuuggcac.......................................................................  1      1   ED5
...................gacggggUuguuuggcacc......................................................................  2      1   ED5
....................acggggUuguuuggcacc......................................................................  12     1   ED5

..................Cgacggggcuguuuggca........................................................................  2      1   EA3
..................ugacggggUuguuuggca........................................................................  1      1   EA3
..................Cgacggggcuguuuggcac.......................................................................  1      1   EA3
..................Cgacggggcuguuuggcacc......................................................................  1      1   EA3
...................gacggggGuguuuggcac.......................................................................  3      1   EA3
...................gacggggUuguuuggcac.......................................................................  4229   1   EA3
...................gacggCgcuguuuggcac.......................................................................  6      1   EA3
...................gacggggcuguuuggcacc......................................................................  3      0   EA3
...................gacggggGuguuuggcacc......................................................................  6      1   EA3
...................gacggggUuguuuggcacc......................................................................  8438   1   EA3
...................gacggggUuguuuggcacca.....................................................................  1      1   EA3
....................acggggGuguuuggcacc......................................................................  1      1   EA3
....................Ccggggcuguuuggcacc......................................................................  1      1   EA3
....................acggggNuguuuggcacc......................................................................  1      1   EA3
....................acggggcuguuuggcacc......................................................................  2      0   EA3
....................acggggUuguuuggcacc......................................................................  18253  1   EA3
....................acggggUuguuuggcacca.....................................................................  3      1   EA3
.....................cggggUuguuuggcacca.....................................................................  1      1   EA3
```
