# Supplementary material for: Exosomal miR-7-25207 Increases Subgroup J Avian Leukosis Virus Titers by Targeting the Akt-CyclinQ1 and PRC1-YAF2 Dual Pathways
Source: Microorganisms. 2024 Jul 22;12(7):1495. doi: 10.3390/microorganisms12071495 (PMC11279298; doi:10.3390/microorganisms12071495)
Supplement: Supplementary file 1 [file Supplemental document S2.pdf › Supplemental document S2.pdf]

A

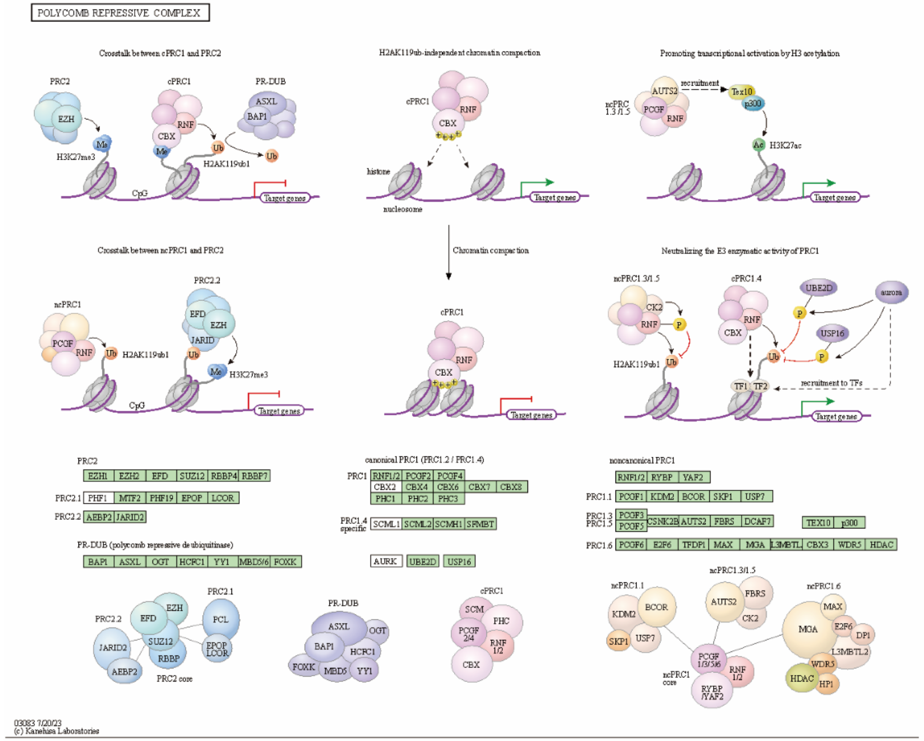
POLYCOMB REPRESSIVE COMPLEX
Crosstalk between cPRC1 and PRC2
H2AK119ub-independent chromatin compaction
Promoting transcriptional activation by H3 acetylation
PRC2
cPRC1
PR-DUB
EZH
ASXL
BAP1
RNF
CBX
Me
Ub
H3K27me3
H2AK119ub1
CpG
Target genes
histone
nucleosome
AUTS2
recruitment
Tex10
p300
PCGF
Ac
H3K27ac
Crosstalk between ncPRC1 and PRC2
Chromatin compaction
Neutralizing the E3 enzymatic activity of PRC1
PRC2.2
ncPRC1
EFD
JARID
UBE2D
aurora
CK2
USP16
recruitment to TFs
TF1
TF2
canonical PRC1 (PRC1.2 / PRC1.4)
noncanonical PRC1
EZH1
EZH2
SUZ12
RBBP4
RBBP7
PRC2.1
PHF1
MTF2
PHF19
EPOP
LCOR
AEBP2
JARID2
PR-DUB (polycomb repressive deubiquitinase)
OGT
HCFC1
YY1
MBD5/6
FOXK
PRC1
RNF1/2
PCGF2
PCGF4
CBX2
CBX4
CBX6
CBX7
CBX8
PHC1
PHC2
PHC3
PRC1.4 specific
SCML1
SCML2
SCMH1
SFMBT
AURK
USP16
RYBP
YAF2
PRC1.1
PCGF1
KDM2
BCOR
SKP1
USP7
PRC1.3
PRC1.5
PCGF3
PCGF5
CSNK2B
FBRS
DCAF7
TEX10
PRC1.6
PCGF6
E2F6
TFDP1
MAX
MGA
L3MBTL
CBX3
WDR5
HDAC
PCL
PRC2 core
SCM
PHC
ncPRC1.1
ncPRC1.3/1.5
ncPRC1.6
DP1
HP1
ncPRC1 core
03083 7/20/23
(c) Kanehisa Laboratories

B

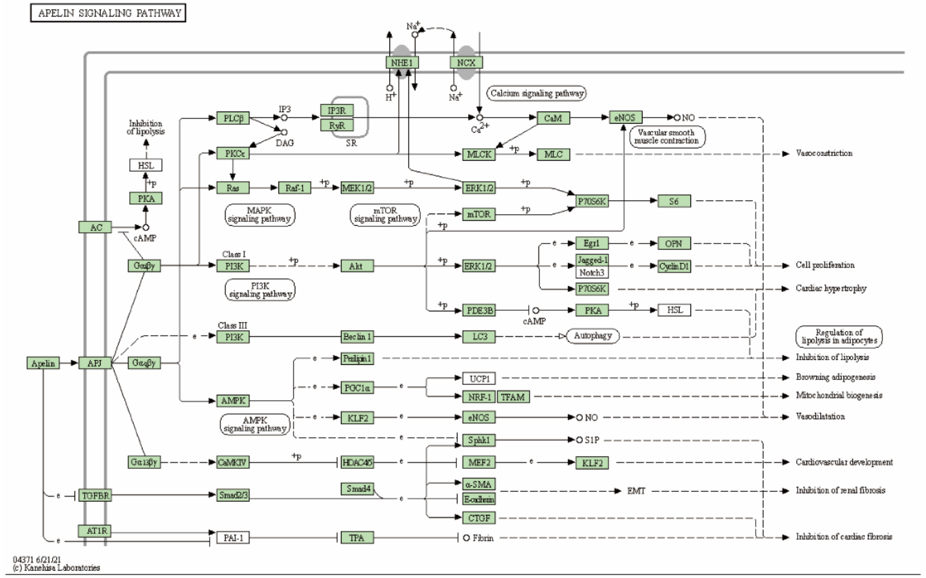
APELIN SIGNALING PATHWAY
Apelin
APJ
AC
cAMP
PKA
HSL
Inhibition of lipolysis
NHE1
NCX
Calcium signaling pathway
PLCβ
IP3
IP3R
RyR
SR
DAG
PKCε
CaM
eNOS
NO
Vascular smooth muscle contraction
MLCK
MLC
Vasoconstriction
Ras
Raf-1
MEK1/2
ERK1/2
P70S6K
S6
MAPK signaling pathway
mTOR
mTOR signaling pathway
Class I
PI3K
Akt
PI3K signaling pathway
Egr1
OPN
Jagged-1
Notch3
CyclinD1
Cell proliferation
Cardiac hypertrophy
PDE3B
Class III
Beclin1
LC3
Autophagy
Regulation of lipolysis in adipocytes
Perilipin1
Inhibition of lipolysis
UCP1
Browning adipogenesis
PGC1α
NRF-1
TFAM
Mitochondrial biogenesis
AMPK
AMPK signaling pathway
KLF2
Vasodilatation
Sphk1
S1P
CaMKIV
HDAC4/5
MEF2
Cardiovascular development
TGFBR
Smad2/3
Smad4
α-SMA
E-cadherin
EMT
Inhibition of renal fibrosis
CTGF
AT1R
PAI-1
TPA
Fibrin
Inhibition of cardiac fibrosis
04371 6/21/21
(c) Kanehisa Laboratories
